# Retrospective Insights of the COVID-19 Epidemic in the Major Latin American City, São Paulo, Southeastern Brazil

**DOI:** 10.3390/v15020327

**Published:** 2023-01-24

**Authors:** Patricia Akemi Assato, Luan Gaspar Clemente, Marta Giovanetti, Gabriela Ribeiro, Alex Ranieri Jeronimo Lima, Melissa Palmieri, Leonardo Nazario de Moraes, Simone Kashima, Heidge Fukumasu, Maurício Lacerda Nogueira, Luiz Carlos Junior Alcantara, Aline Lais Nicolodelli, Antonio Jorge Martins, Bruna Petry, Cecilia Artico Banho, Claudia Renata Dos Santos Barros, Cristina Tschorny Moncau-Gadbem, Debora Botequio Moretti, Debora Glenda Lima De La Roque, Elaine Cristina Marqueze, Elisangela Chicaroni Mattos, Fabiana Erica Vilanova Da Silva, Felipe Allan Da Silva Da Costa, Giselle Cacherik, Jardelina De Souza Todao Bernardino, Jessika Cristina Chagas Lesbon, Lívia Sacchetto, Loyze Paola Oliveira De Lima, Luiz Artur Vieira Caldeira, Maiara Martininghi, Marília Mazzi Moraes, Mirele Daiana Poleti, Pedro De Queiroz Cattony Neto, Raquel De Lello Rocha Campos Cassano, Ricardo Augusto Brassaloti, Svetoslav Nanev Slavov, Vincent Louis Viala, Luiz Lehmann Coutinho, Rejane Maria Tommasini Grotto, Raul Machado Neto, Dimas Tadeu Covas, Sandra Coccuzzo Sampaio, Maria Carolina Elias, Jayme A. Souza-Neto

**Affiliations:** 1 School of Agricultural Sciences, São Paulo State University (UNESP), Botucatu 18610-034, Brazil; 2 Centro de Genômica Funcional da ESALQ, University of São Paulo, Piracicaba 13418-900, Brazil; 3 Instituto Oswaldo Cruz, FIOCRUZ, Rio de Janeiro 21040-360, Brazil; 4 Instituto de Ciências Biológicas, Universidade Federal de Minas Gerais, Belo Horizonte 31270-901, Brazil; 5 Department of Science and Technology for Humans and the Environment, University of Campus Bio-Medico di Roma, 00128 Rome, Italy; 6 Butantan Institute, Sao Paulo 05508-040, Brazil; 7 Health Surveillance Coordination, Sao Paulo Municipal Health Department—Coordenadoria de Vigilância em Saúde—Secretaria Municipal de São Paulo, Sao Paulo 05579-000, Brazil; 8 Ribeirão Preto Medical School, Blood Center of Ribeirão Preto, University of São Paulo, Ribeirão Preto 14051-140, Brazil; 9 Department of Veterinary Medicine, School of Animal Science and Food Engineering, University of Sao Paulo, Pirassununga 13635-900, Brazil; 10 Faculdade de Medicina de São José do Rio Preto (FAMERP), São José do Rio Preto 15090-000, Brazil; 11 Primary Care Coordination/Sao Paulo Municipal Health Department—Coordenadoria de Atenção Básica—Secretaria Municipal de São Paulo, Sao Paulo 05579-000, Brazil; 12 Genomic Surveillance Network, São Paulo State University (UNESP), Sao Paulo 01049-010, Brazil

**Keywords:** COVID-19, SARS-CoV-2, genomic surveillance, São Paulo, Brazil

## Abstract

São Paulo is the financial center of Brazil, with a population of over 12 million, that receives travelers from all over the world for business and tourism. It was the first city in Brazil to report a case of COVID-19 that rapidly spread across the city despite the implementation of the restriction measures. Despite many reports, much is still unknown regarding the genomic diversity and transmission dynamics of this virus in the city of São Paulo. Thus, in this study, we provide a retrospective overview of the COVID-19 epidemic in São Paulo City, Southeastern, Brazil, by generating a total of 9995 near-complete genome sequences from all the city’s different macro-regions (North, West, Central, East, South, and Southeast). Our analysis revealed that multiple independent introduction events of different variants (mainly Gamma, Delta, and Omicron) occurred throughout time. Additionally, our estimates of viral movement within the different macro-regions further suggested that the East and the Southeast regions were the largest contributors to the Gamma and Delta viral exchanges to other regions. Meanwhile, the North region had a higher contribution to the dispersion of the Omicron variant. Together, our results reinforce the importance of increasing SARS-CoV-2 genomic monitoring within the city and the country to track the real-time evolution of the virus and to detect earlier any eventual emergency of new variants of concern that could undermine the fight against COVID-19 in Brazil and worldwide.

## 1. Introduction

In December 2019, a respiratory infection outbreak was first reported in Wuhan, Hubei Province, China, among workers from the Huanan Wholesale market, and by the end of January 2020, it quickly reached most of the countries in the world [1,2].

The illness, found to be caused by severe acute respiratory syndrome coronavirus 2 (SARS-CoV-2), a betacoronavirus with a genome of ~30 kb [3], was named coronavirus disease 19 (COVID-19), and was further declared a pandemic on 11 March 2020. Since then, COVID-19 has been considered the major public health concern in the world, resulting in more than 500 million cases and 6 million deaths worldwide, with Brazil holding one of the highest numbers of cases and deaths (WHO dashboard).

**Figure 1:**
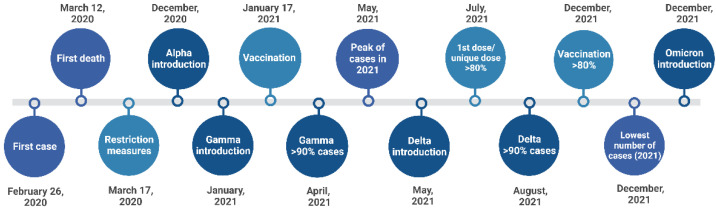
Summary of main events of the COVID-19 epidemic in São Paulo City.

The first case of COVID-19 in Brazil was reported in São Paulo City on 26 February 2020, in a 61-year-old male who had recently returned from Italy. The first death in the country, a 57-year-old female without a traveling history, was also reported in São Paulo City two weeks later, confirming that local COVID-19 community transmission was taking place in the city [4,5]. A couple of days later, on 16 March 2020, after an expressive increase in the number of reported cases, an emergency state was instituted in the city, triggering the implementation of several restriction measures to contain the dissemination of SARS-CoV-2 [6]. At the beginning of the COVID-19 pandemic, São Paulo City was considered a quick-spread city and to date presents the highest number of cases and deaths reported in the country, 1,124,958 and 43,975, respectively [7,8,9]. A timeline of the main events concerning the COVID-19 epidemic in São Paulo City is summarized in Figure 1.

**Figure 2:**
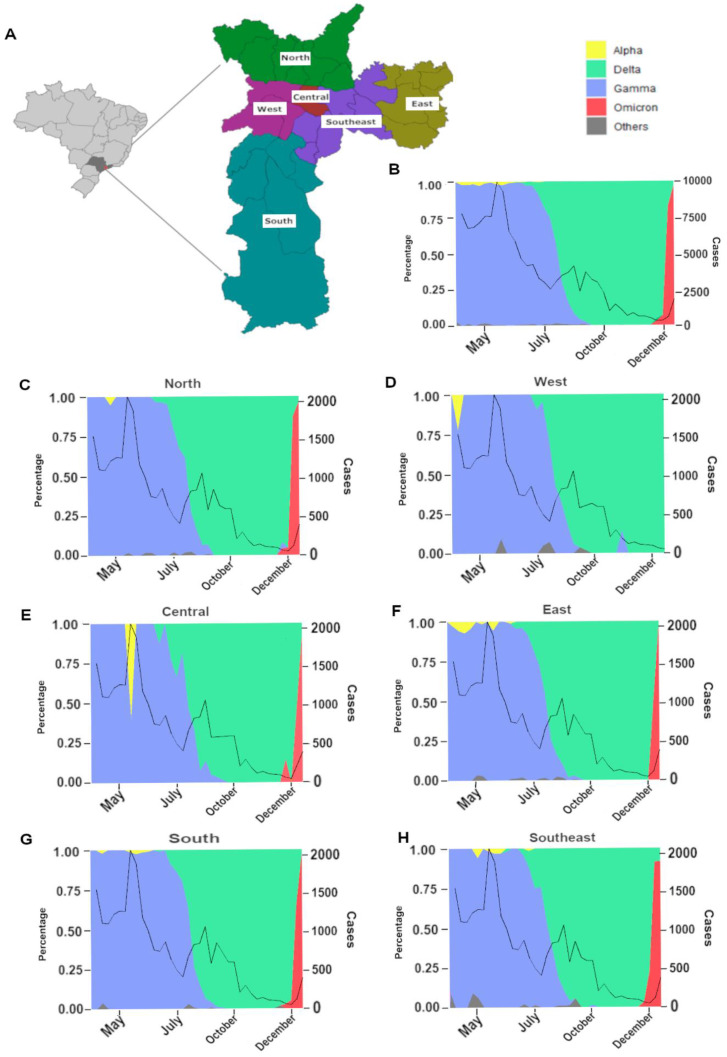
Dynamics of the SARS-CoV-2 epidemic in the city of São Paulo, Southeast Brazil. (**A**) Map of Brazil highlighting São Paulo City, colored by its regions. Distribution of SARS-CoV-2 variants from March to December 2021 in São Paulo City (**B**) and by the city’s regions: North (**C**), West (**D**), Central (**E**), East (**F**), South (**G**), and Southeast (**H**).

São Paulo City, the most populated city in Latin America with approximately 12 million citizens, is considered the financial center of Brazil. The city is divided into six regions: Central, North, West, East, Southeast, and South (Figure 2A), which are highly interconnected by an extensive public transportation network that includes buses, light rails, and subways. São Paulo City also has three major bus stations (Tiete, Barra Funda, and Jabaquara) that receive passengers from all over Brazil and many neighboring South American countries, and a busy domestic airport (Congonhas Airport). In addition, the Greater São Paulo (which is encompassed by São Paulo City and its surrounding municipalities) is home to the GRU International Airport, the major airport in South America and the second busiest one in Latin America.

The emergence of new SARS-CoV-2 variants in late 2020 raised new concerns about how these variants may be able to affect the course of the COVID-19 pandemic and reinforced the importance of genomic surveillance that allows not only the monitoring of the circulating variants, but also detects new SARS-CoV-2 variants with mutations related to pathogenesis, transmissibility, evasion of host immune response, and vaccine effectiveness [10,11,12,13]. In addition, genomic epidemiology studies have been useful to demonstrate the importance of restriction measures, evaluate the effect of different VOCs in the course of the COVID-19 pandemic in different waves, and contribute to future epidemiological studies and public health measures [14,15,16].

In Brazil, a great effort was made to establish networks and infrastructure for SARS-CoV-2 genomic surveillance, which resulted in a better understanding of the pandemic dynamics in the country [4], and allowed the identification of new variants and sublineages [17,18]. São Paulo State has the highest number of SARS-CoV-2- sequenced samples in Brazil [4]. In 2021, the São Paulo State Network for SARS-CoV-2 Variants Alert was implemented to monitor the variants across the state, and to generate weekly epidemiological reports on the COVID-19 epidemic in the state [19].

The understanding of the COVID-19 epidemic in São Paulo City could shed light on the local genomic diversity and transmission dynamics of SARS-CoV-2 in this major economic and transportation hub in South America. Hence, we retrospectively investigated the COVID-19 epidemic scenario in the city of São Paulo. To the best of our knowledge, this is the major genomic epidemiology study of SARS-CoV-2 variants in a Brazilian metropolis and could bring insights to future epidemiological studies.

## 2. Materials and Methods

### 2.1. Ethical Statement

The study was conducted according to the guidelines of the Declaration of Helsinki and approved by the Institutional Ethics Committee of the Faculty of Medicine of Ribeirão Preto, University of São Paulo (CAAE: 50367721.7.1001.5440).

### 2.2. Clinical Samples

Samples from positive cases for COVID-19 were randomly selected by the São Paulo State Network for SARS-CoV-2 Variants Alert for whole genome sequencing. Samples were selected for sequencing based on the Ct value (≤30) and availability of epidemiological metadata, such as date of sample collection, sex, age, and the municipality of residence. The preparation of SARS-CoV-2 genomic libraries was performed using the Illumina COVIDSeq kit following the manufacturer’s instructions using the ARTIC Network PS (https://github.com/artic-network/artic-ncov2019/tree/master/primer_schemes/nCoV-2019/V3 accessed on 12 January 2021). The normalized libraries were loaded onto a 300-cycle MiSeq Reagent Kit v2 and run on the Illumina MiSeq instrument (Illumina, San Diego, CA, USA). For convenience, the geographical locations were aggregated by the São Paulo City macro-regions: North, West, Central, East, South, and Southeast (Table S1).

### 2.3. Consensus Sequences

The raw sequence data obtained were analyzed in FASTQC (ref) software version 0.11.8 for quality control analysis followed by trimming, to select the sequences with the best quality (quality score > 30), using Trimmomatic [20] version 0.3.9. The trimmed sequences were mapped against the SARS-CoV-2 reference genome (Genbank refseq NC_045512.2) using BWA [21] software and SAMtools [22] for read indexing. The refinement of mapped sequences was performed in Pilon software [23], to obtain the most reliable deletions and insertions. The trimmed sequences were remapped against the genome improved by Pillon [23]. We then used bcftools [24] for variant calling, and seqtk (https://github.com/lh3/seqtk accessed on 20 February 2021) for assembling the consensus genomes. Positions covered by fewer than 10 reads (DP < 10) and bases whose quality was lower than 30 were considered a gap in coverage and converted to Ns. Coverage values for each genome were calculated using SAMtools v1.12 [16]. The quality of the consensus genome sequences was finally assessed using Nextclade [25].

### 2.4. Phylogenetic and Phylodynamic Analysis

To ensure the quality of the genome sequences generated in this study and to guarantee the highest possible phylogenetic accuracy, only genomes >29,000 bp and <1% of ambiguities were considered (n = 9995). We appended the 9995 genome sequences newly generated under this project with an extensive reference data set of SARS-CoV-2 sequences sampled globally, including n = 68,194 near-complete genomes from Brazil and n = 17,072 from worldwide, sampled up to December 2021 (Table S2). For downsampling, we applied a cluster approach based on 99.9% of identity between samples (http://weizhong-cluster.ucsd.edu/cd-hit/ accessed on 20 January 2022), retrieving a total of n = 16,357 near-complete genomes from Brazil and n = 16,797 from worldwide.

Phylogenetic analysis was performed according to Giovanetti et al. [4]. Briefly, sequences were aligned using Nextalign [25] and submitted to IQ-TREE 2 [26] for maximum likelihood phylogenetic analysis. Our data set was also assessed for both sequences with low data quality (e.g., with assembling issues, sequencing and alignment errors, data annotation errors, and sample contamination) and for molecular clock signal (i.e., temporal structure) using TempEst v1.5.3 [27]. The raw ML tree topology was then used to estimate the number of viral transmission events between various Brazilian regions and the rest of the world. TreeTime [28] was used to transform this ML tree topology into a dated tree using a constant mean rate of 8.0 × 10^−4^ nucleotide substitutions per site per year, after the exclusion of outlier sequences. A migration model was fitted to the resulting time-scaled phylogenetic tree in TreeTime, mapping country and regional locations to tips and internal nodes [28]. Using the resulting annotated tree topology, we were able to evaluate the transition rate (i.e., virus importations and exportations) between different regions of São Paulo City. Importantly, this analysis was not dependent on a monophyletic clustering of SARS-CoV-2 within the city of São Paulo.

## 3. Results

### SARS-CoV-2 Variants in São Paulo City

In this study, we analyzed a total number of 9995 near-complete SARS-CoV-2 genome sequences from confirmed COVID-19 patients from the city of São Paulo. The samples were collected between April and December 2021, from the following regions: Central (367 sequences), North (2392 sequences), West (606 sequences), East (2548 sequences), Southeast (1910 sequences), and South (2656 sequences), and made available to the Network for Pandemic Alert of Emerging SARS-CoV-2 Variants of the State of São Paulo for sequencing.

The analysis of SARS-CoV-2 variants distribution in the city of São Paulo and per region in 2021 (Figure 2B–H) revealed a similar distribution of cases throughout time. Furthermore, it highlighted the crucial role of the Gamma and the Delta VOCs in the progression of the SARS-CoV-2 epidemic across the city’s macro-regions during the second and the third epidemic wave, respectively. The second wave of COVID-19 was promoted by the introduction of the Gamma VOC at the beginning of 2021, which represented almost 99% of the variants identified by April 2021 in the city of São Paulo. During this wave, São Paulo had the highest number of COVID-19 cases and associated deaths [8].

In June 2021, the first cases of the Delta VOC started to be reported in the state and since then this VOC became the most prevalent one, which rapidly spread within all the macro-regions in São Paulo City and within the country. By the end of August 2021, it represented most of the sequenced samples, ranging from 67 to 73% (Figure 2C–H). An increase in reported cases was observed early in July. However, the number of cases started to rapidly decrease, in all regions, in September 2021, probably due to the reasonable percentage of the population with natural immunity acquired by prior infection with Gamma together with the number of vaccinated people (Figure 2B–H).

The Omicron VOC was introduced in São Paulo in early December 2021 and rapidly spread across the city with a significant increase in the number of cases reported. By the end of December 2021, Omicron represented 45% of sequenced samples in the city, with a higher prevalence in North (Figure 2C) and Central regions (Figure 2E), 63% and 73%, respectively. Omicron was not detected by genomic surveillance only in the West region, which had a later spread of that VOC (Figure 2D).

The Alpha VOC, which was already considered a variant of concern at the time, was detected in different periods in each region during the second wave. However, it did not become dominant in the city, likely due to the upcoming introduction and rapid spread of Gamma. Nonetheless, a higher frequency of the Alpha VOC could be observed in the Central region of São Paulo City during May 2021 (Figure 2E).

**Figure 3:**
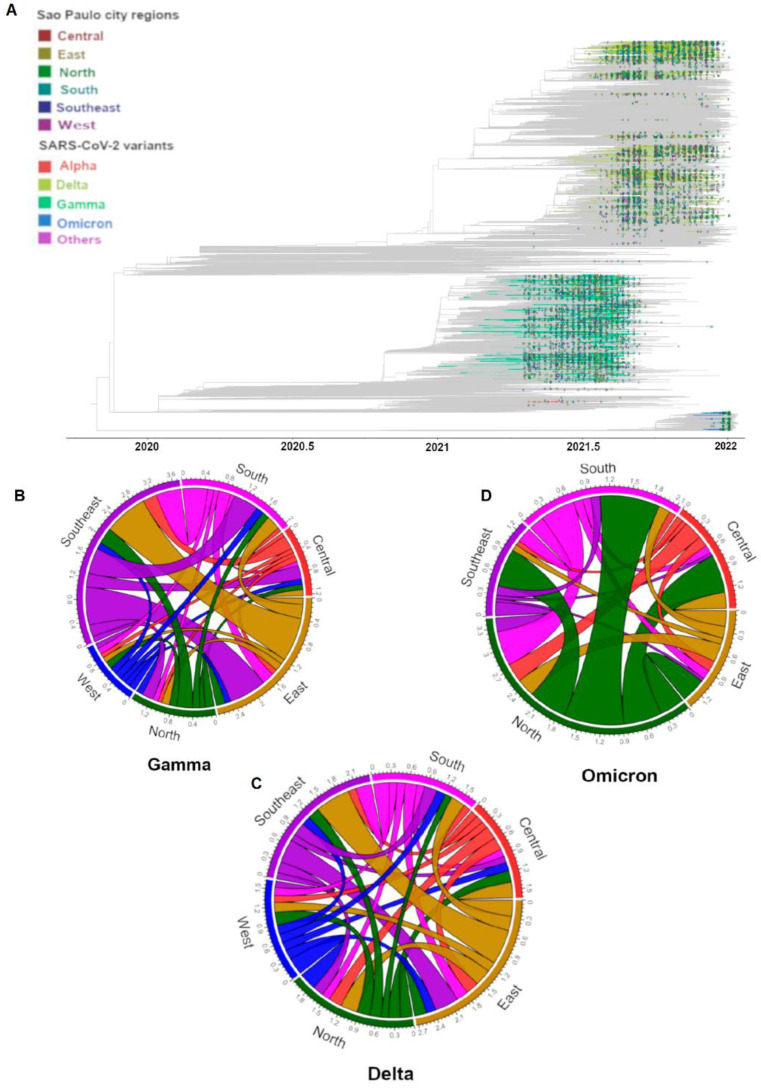
Phylogenetic analysis and SARS-CoV-2 lineage dynamics in the city of São Paulo, Southeast Brazil. (**A**) Time-resolved maximum likelihood phylogeny containing high-quality near-full-genome sequences from the city of São Paulo (n = 9995) obtained from this study, and analyzed against a backdrop of global reference sequences (n = 33,154). Variants of concern (VOC) are highlighted on the phylogeny. (**B**–**D**) Number of viral exchanges within São Paulo macro-regions by counting the state changes from the root to the tips of the phylogeny in panel (**B**–**D**).

In order to evaluate the dispersion of the Gamma, Delta, and Omicron VOCs across the regions of São Paulo City, a phylogenetic analysis was performed. For this, in addition to genome sequences generated in our study, we retrieved worldwide SARS-CoV-2 genome sequences from GISAID, which were used for tree reconstruction. Our analysis revealed that multiple independent SARS-CoV-2 introduction events mediated the progression of those variants within all the macro-regions of the city of São Paulo (Figure 3A), which likely spread through regional mobility pathways during distinct epidemic waves.

Using an ancestral location state reconstruction on the dated phylogeny, we were able to infer the number of viral imports and exports among all the macro-regions in the city of São Paulo (Figure 3B–D). Our analysis revealed that although the dispersion happened between all regions, the East and Southeast regions were the largest contributors of viral movements from one geographical region to another regarding Gamma and Delta variants, respectively (Figure 3B,C). Meanwhile, the North region had a higher contribution in the dispersion of the Omicron variant within the city (Figure 3D). The transition rates between pairs of regions can be found on Supplementary Table S3.

## 4. Discussion

Genomic surveillance has been a powerful tool to monitor and detect variants and can be helpful in the control of the SARS-CoV-2 dissemination in both local and large scales as has been demonstrated in several studies [14,29,30]. For example, Dellicour et al. [31] evaluated the dynamics of the first COVID-19 wave in New York City, and demonstrated the epidemiological importance of Queens during this period as a transmission hub that played an important role in the viral dispersion.

In this study, we evaluated the frequency and dispersion of SARS-CoV-2 variants along 2021 within and among the legal regions of São Paulo City (Central, North, West, East, Southeast, and South). Our results show the introduction and subsequent replacement of SARS-CoV-2 VOCs in São Paulo regions during 2021. The second wave of COVID-19 in São Paulo was due to the introduction of the Gamma variant, which was originated in Manaus, Brazil in January 2021 and disseminated all over the country [29]. São Paulo City had the highest number of cases and deaths for that period, and the Gamma variant represented more than 90% of the sequenced genomes from all regions. 

COVID-19 vaccination started in São Paulo on 17 January 2021, and due to limited dose availability was carried out in phases considering the target population’s age and risk of SARS-CoV-2 infection. By the end of May 2021, which corresponds to the peak of COVID-19 in the city, around 25% of the adult population had received a first vaccine dose, whereas some 12.5% were fully vaccinated with two doses [30].

In August 2021, the replacement of the Gamma by the Delta variant took place in all regions of São Paulo City, resulting in a temporary increase in COVID-19 cases just after the introduction of Delta. However, this trend was reversed in the following months with the number of cases decreasing again and reaching its lowest levels at the beginning of December 2021. Hence, the introduction of the Delta variant in São Paulo, which was initially seen with great concern due to its increased transmission potential [1], did not result in a consistent increase of COVID-19 cases in the city. This is presumably due to the concomitant and progressive increase in vaccination coverage in the city, where more than 35% and 75% of its population, respectively, had received full and partial vaccination at that time [30]. 

In late November 2021, Omicron was identified and raised great concern worldwide due to the number of mutations and the rapid spread in Southern Africa [31]. In early December 2021, the first cases were reported in Brazil, including those in São Paulo City, and by the end of the year, Omicron had replaced Delta in some regions of São Paulo, such as Center and North. Besides, the number of COVID-19 cases started to increase, even though at this time over 80% of its adult population had the vaccination scheme completed and about 20% had received the additional dose.

São Paulo is highly connected by a busy public transportation system that allows massive mobility across the city and can directly contribute to virus dissemination. During the Gamma and Delta waves, we could observe a similar pattern in the dispersion of these variants among all regions, especially from East and Southeast regions to other regions. During Omicron spread, the North region exerted a higher contribution in viral dispersion, which could be associated with a higher circulation of people from and to other cities due to the end of the year holidays. In addition, the Northern region harbors one of the busiest bus stations in the country, the Tiete Bus Station, and also the adjacent GRU International Airport, the major international airport in South America. According to the São Paulo tourism research center, in December 2021, São Paulo’s bus stations registered 42.1% and São Paulo airports (GRU, Congonhas and Viracopos) registered a 35.9% increase in passenger flow compared to September 2021 [32]. That could explain the change in Omicron dispersion between the regions, as the North region seems to have a higher influence in the spread when compared to other regions. 

## 5. Conclusions

In this study, we presented the evolution of SARS-CoV-2 variants throughout time in São Paulo City, which reinforces the importance of genomic surveillance for monitoring the real-time evolution of SARS-CoV-2 variants and the active monitoring of these variants. Understanding their transmission dynamics is crucial to mitigate disease transmission. In addition, São Paulo has the potential to be a super-spreader city due to the high circulation of local/surrounding citizens and domestic/international tourists. Therefore, public health strategies to prevent the transmission of communicable/airborne pathogens should be increased in high- movement areas, such as airports and bus stations.

## Data Availability

All sequences that were generated and used in the present study are listed in Table S2 along with their GISAID sequence IDs, dates of sampling, the originating and submitting laboratories, and main authors.

## Supplementary Materials

The following supporting information can be downloaded at: https://www.mdpi.com/article/10.3390/v15020327/s1, Table S1: Sequenced samples by São Paulo regions; Table S2: SARS-CoV-2 genomes; Table S3: Transition rates between regions.
